# Liquid metal capsules for autonomic thermal energy control

**DOI:** 10.1039/d6ra02315k

**Published:** 2026-05-19

**Authors:** Bo Yu, Hongqiang Wang, Elena M. Shchukina, Mikhail Zheludkevich, Mathew Quarrell, Bernard P. Binks, Dmitry G. Shchukin

**Affiliations:** a Stephenson Institute for Renewable Energy, University of Liverpool Liverpool L69 7ZF UK d.shchukin@liverpool.ac.uk; b Lanzhou Institute of Chemical Physics Lanzhou 730000 P. R. China; c School of Materials Science and Engineering, Northwestern Polytechnical University Xi'an 710072 P.R. China; d Helmholtz-Zentrum Hereon, Institute of Surface Science Max-Planck-Straße 1 21502 Geesthacht Germany; e Department of Chemistry, University of Hull Hull HU6 7RX UK

## Abstract

This research is focused on the ultrasonic encapsulation of low temperature GaIn eutectic alloy using silica nanoparticles as a Pickering shell. GaIn eutectic has a solid/liquid transition temperature at 15.7 °C and can be used for autonomic heat uptake and release as a phase change material (PCM). The best solvent for ultrasonic encapsulation of GaIn is chloroform resulting in capsules of 1.4 µm possessing a silica nanoshell which are stable for at least 5 years. Differential scanning calorimetry confirmed the heat uptake/release performance during at least 50 thermal cycles. GaIn capsules were incorporated into porous Al/Mg light weight alloy used in the aerospace industry for autonomic control of its surface temperature. Capsules demonstrated an effective decrease of the alloy surface temperature from 82 °C to 30 °C during infrared heating of the samples. Compared to other PCMs, encapsulated liquid metals have the advantages of high electric and thermal conductivity, low melting temperature and immiscibility with usual solvents. They can be used as autonomic micro and nano containers in aerospace or electronic industries.

## Abstract 2

This research proposed the use of ultrasound for the encapsulation of liquid metal eutectic GaIn into a silica Pickering shell. Eutectic capsules can be used for autonomic thermal control in chips, batteries and photovoltaic cells.

## Graphical abstract

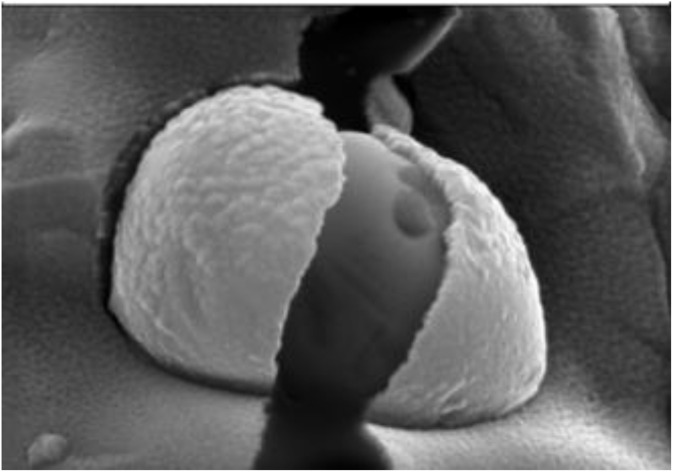

## 1 Introduction

Common organic PCMs include paraffins, fatty acids, fatty acid esters and polyethylene glycols. Paraffins are non-toxic solid–liquid PCMs, and the melting temperature increases with the number of carbon atoms.^1^ They exhibit high thermal reliability and cycling stability. Fatty acids have the general formula of CH_3_(CH_2_)_2*n*_COOH. They have a high latent heat of fusion, almost no supercooling, no phase separation and good cycling stability. However, they are corrosive.^2^ Polyethylene glycol is a polyether terminated with a hydroxyl group expressed as H(OC_2_H_4_)_*n*_OH. Polyethylene glycol shows good chemical and thermal stability, non-toxicity, non-corrosivity and biodegradability but glycol-based PCMs are not conductive, and their application is mostly limited to the construction industry. Inorganic PCMs include salts, hydrated salts and metals. They could have broad applications as their melting points span from the low temperature range with hydrated salts to the high temperature range of several hundred °C with salts and metals.^3^ The main drawback of salts and hydrated salts is that they are very corrosive and cannot be used as PCMs for temperature control of metal structures.^4^

Liquid metals, which are impregnated into semiconducting and/or insulating coatings, are believed to offer important opportunities for electronic and electrochemical devices considering the formed metal–semiconductor junctions and/or the circumventing of direct contact between circuit and liquid metal.^5^ The past efforts revealed the success of macroscopically sized liquid metals in applications such as electronic printing,^6^ while micro/nanoscaled liquid metals would be of greater interest due to their potential use in emerging miniaturised devices. Mercury micro-rods were used to make an electromagnetic meta-material operating at microwave wavelengths.^7^ Stretchable micro-antennas were fabricated from gallium–indium–tin (GaInSn) and GaIn eutectic alloys in dielectric materials.^8,9^ Metal droplets found applications in optical switches, pumps and valves in electromechanical systems for high-speed operations, *e.g*. in electronics and transportation.^10,11^

Metals such as Al, Cu, Mg and Zn^12,13^ can be fused to metallic alloys with large latent heat density and good thermal stability for high temperature PCM applications with fixed melting temperatures and enthalpies. Metal eutectics, on the other hand, have a wide range of melting temperatures and enthalpies depending on the content of the metals in the eutectic. AlCu eutectic alloy demonstrated microstructure consisting of fine and coarse Al + Al_2_Cu eutectic regions with a latent heat of 319.5 J g^−1^.^14^ Two binary eutectic alloys Mg_84_Cu_16_ and Mg_59_Cu_41_ were studied for their structural, thermophysical and corrosion performance.^15^ The melting point and latent heat of Mg_84_Cu_16_ are 488 °C and 232 J g^−1^, respectively, while they are 550 °C and 138 J g^−1^ for Mg_59_Cu_41_. Magnesium–copper alloys have high thermal conductivity in the temperature range 400–550 °C. The microstructure of MgSn alloy mainly consisted of α-Mg matrix and α-Mg + Mg_2_Sn eutectic phases.^16^ The melting enthalpies of Mg–24% Sn, Mg–37% Sn and Mg–50% Sn alloys are 105.3, 217.8 and 118.8 J g^−1^, with phase change temperatures of 557.6, 554.4 and 557.1 °C, respectively.

However, there is another important application of liquid metals as PCMs for thermal energy storage at low temperatures (below 50 °C) for passive cooling of electronic devices. In general, PCMs can provide thermal management in an efficient and elegant way. The heat produced in industrial and domestic sectors can be collected and redistributed, so the temperature difference between different parts of, *e.g*. batteries or electric devices, can 0062e compensated. Liquid metal eutectics with low temperature melting points are used in lithium-ion batteries to control optimal operating temperature during charge uptake and release, *e.g*. BiInSn alloy with 60 °C melting point and 27.9 J g^−1^ latent heat.^17^ Together with high thermal conductivity and zero corrosion activity, BiInSn alloy can be used in heat sink technology.^18^ However, the problem of the stabilization of liquid metal droplets against leakage, coalescence and reaction with the local environment (ink formulation, coating matrix) requires solutions for isolation of the liquid metal core. The encapsulation of micro and nanosized metal eutectics within a robust and protective shell can provide the required level of protection retaining at the same time thermal and electrical properties of the liquid metal core.

There are several microfluidic approaches reported in the literature for the formation of micro-droplets of liquid metal eutectics at room temperature. Gallium–indium liquid metal micro-droplets, both spherical and non-spherical, were formed at room temperature in microfluidic devices filled with either aqueous polyethylene glycol solution or oxygenated silicone oil.^19^ Those in water required the addition of a surfactant to prevent their coalescence whereas those in oil did not; the instantaneous formation of a rigid oxide skin on the alloy surface was deemed responsible for droplet stabilisation. The volume of the droplets depends on the channel dimensions and flow rates applied, varying between 0.5 and 4 nL. The addition of polyvinyl alcohol or polymethylmethacrylate stabilises the emulsions of liquid metal prepared and also leads to a decrease in the droplet size.^20^ Micro-droplets of liquid metals in all microfluidic studies were mechanically stabilised only by the native oxide layer.^21^ This layer is so thin that it does not alter significantly the electrical properties of the alloy^22^ but can be destroyed by application of a small external force.^23^ Therefore, formation of a robust capsule shell around the metal core is necessary for the protection of the dispersed liquid metal.

Small colloidal particles can be surface-active at an oil-water interface and can stabilize Pickering emulsions.^24^ The adsorbed particle layer around dispersed droplets provides a steric barrier to coalescence with ultra-stable emulsions being formed. We demonstrate in this paper a general ultrasonic approach to prepare GaIn eutectic microcapsules in solvents with different polarity stabilized by fumed silica nanoparticles of controlled hydrophobicity on their surfaces. We studied the influence of the confined volume of liquid metal microcapsule on the liquid/solid phase thermal transition characteristics demonstrating encapsulated low temperature liquid metal eutectics for autonomic temperature control of the lightweight Al/Mg macroscopic alloys.

## 2 Materials and methods

### 2.1 Materials

Water was passed through a reverse osmosis unit and then a Milli-Q reagent water system for purification. Chloroform and cyclohexane were purchased from Sigma-Aldrich (99% purity). GaIn (75.5:24.5 wt%) eutectic alloy from Alfa Aesar was used as an example of the liquid metal with a bulk melting point of 15.7 °C (Alfa Aesar data). It has a surface tension around room temperature of >600 mN m^−1^ and a density of 6.2 g cm^−3^. Fumed silica nanoparticles (primary diameter 20–30 nm, amorphous, Wacker Chemie) were modified with dimethyldichlorosilane^25^ and used to stabilise liquid metal-in-solvent droplets as Pickering emulsions. Hydrophilic silica was silylated to various extents by reaction with dimethyldichlorosilane in the presence of water followed by drying at 300 °C for 2 h leaving the particle surfaces containing silanol (SiOH) and dimethylsilane groups. The silanol content was determined by titration with aqueous NaOH. The wettability of the particles was characterised in terms of the percentage of unreacted SiOH groups on surfaces. It ranged from 100% (most hydrophilic) to 15% (most hydrophobic). Thermal conductivity of amorphous silica nanoparticles in Pickering eutectic capsules is 0.5 W (m K^−1^) according to DSC measurements.

### 2.2 Encapsulation process

Encapsulation of the liquid metal alloy within the silica particle shell was performed using high intensity ultrasound both for dispersion of the liquid metal droplets and formation of the nanoparticle shell. A typical emulsion contains silica, GaIn alloy and solvent. GaIn alloy is the inner dispersed droplet phase, and solvent is the outer continuous phase. Ultrasound is a very powerful technique for dispersion (down to 50 nm size) and simultaneous encapsulation of immiscible liquids either in polymer capsules or in capsules containing dispersed nanoparticles in the shell.^26^ First, the silica particles were dispersed in the solvent using a Qsonica Q700 sonicator operating at 20 kHz and 140 W for 2 min with 1/2 inch ultrasonic horn, and then GaIn eutectic alloy was added to the silica dispersion in the desired quantity and sonication continued for another 1 min. All ultrasonic treatments were performed at room temperature in open air conditions. We examined three different solvents: water (dielectric constant, *ε* = 80), chloroform (*ε* = 5) and cyclohexane (*ε* = 2) as the continuous phase for formation of Pickering emulsions to study the influence of solvent polarity on the stability and thermal properties of the resulted encapsulated eutectic. GaIn capsules were incorporated into anodised porous Al/Mg alloy (average pore size is 2.4 µm) by dipping into eutectic capsules from chloroform Pickering emulsion containing 20 mg mL^−1^ GaIn and 30 mg mL^−1^ silica particles possessing 50% SiOH on the surface for 20 min with achieve maximum 2 wt% incorporation efficiency of GaIn eutectic capsules.

### 2.3 Characterisation

Optical imaging was done using an optical microscope equipped with a Canon EOS 1100D Digital SLR camera with a 12-megapixel APS-C-sized sensor. An internal reference ruler was used for the reference line and Image J software was used to determine the diameter of the capsules. SEM and cryo-SEM analysis was performed using a JEOL S-4800 instrument (Hitachi). Thermal imaging (FLIR T640, sensitivity <0.03 °C) was used to monitor Al/Mg metal alloy surface temperature with and without eutectic capsules during thermal cycling. The FLIR camera was housed in a sealed metal box (53 × 65 × 76 cm) which was lined with cork to prevent reflection of the IR interfering with the measurement and to prevent thermal interference from the environment. Each sample was heated under a 150 W Phillips infrared lamp for 5 min at 20 cm distance and then allowed to cool to room temperature for 30 min after switching-off infrared lamp. Thermal images were taken every 15 s. This was repeated three times with mass measurements of the samples taken before and after. No significant mass loss was observed. The energy storage properties, stability of the GaIn capsules during heat uptake and release and melting temperature were determined using differential scanning calorimetry (DSC 214 from Netzsch, Germany) between −15 °C and +30 °C at a rate of 2 °C min^−1^ in perforated aluminium pan under nitrogen flow. Each measurement contained 10 mg of sample for precise measurements above detection limits.

## 3 Results and discussion

No stable eutectic capsules in cyclohexane were achieved at any volume fraction of GaIn or silanol content (Fig. S1). Particles gelled in non-polar solvent even at low % SiOH (Fig. S2).

**Fig. 1:**
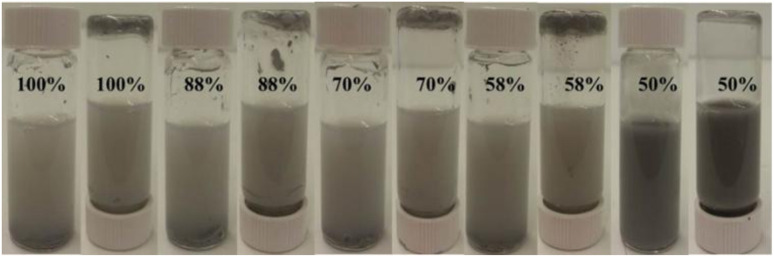
Photos of 10 mL vials containing liquid eutectic capsules in water after 1 day with 20 mg mL^−1^ GaIn and 30 mg mL^−1^ silica particles with different SiOH content (given) in 6 mL of water. The top of the inverted vials contains aggregates of precipitated metal droplets.

The ability to encapsulate liquid eutectic and form stable Pickering emulsions is improved in polar solvents compared to cyclohexane. Silica nanoparticles with SiOH content of 50% and higher can be dispersed in water with ultrasound (Fig. S3) forming eutectic capsules. The best stability in water against both coalescence and sedimentation was found for particles possessing 50% SiOH (Fig. 1). Samples with higher SiOH content (58–100%) also formed eutectic capsules in water but with lower stability and formation of a gelled structure on the bottom of the vial. An increase in the concentration of silica particles or a decrease in the volume fraction of GaIn alloy leads to an improvement in emulsion stability (Fig. S4 and S5). The emulsion in water containing 20 mg mL^−1^ GaIn and 30 mg mL^−1^ silica nanoparticles with 50% SiOH is stable for 8 days before sedimentation. The average diameter of the eutectic capsules in water is 2.2 µm with a polydispersity index of 12%.

**Fig. 2:**
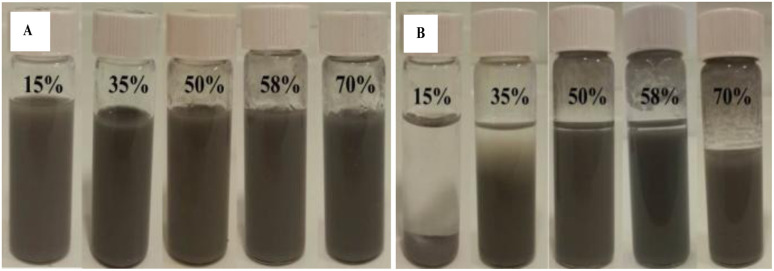
Photos of (A) freshly prepared eutectic capsules in chloroform and (B) after 8 days containing 20 mg mL^−1^ GaIn eutectic alloy and 30 mg mL^−1^ silica particles with different % SiOH on their surface.

The most stable eutectic capsules were obtained in chloroform, the solvent with an intermediate polarity between cyclohexane and water. As seen in Fig. 2, emulsions containing 30 mg mL^−1^ silica particles possessing 15% and 35% SiOH on their surface are unstable and sediment after 8 days. Eutectic capsules with 50% SiOH content disperse in chloroform uniformly, higher SiOH content of 58% and 70% results in a gelled structure at the same concentration which can be observed at the top of the liquid.

**Fig. 3:**
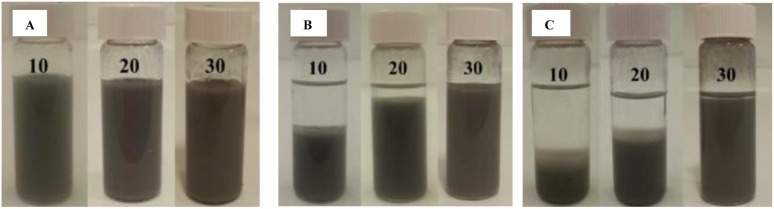
Photos of (A) freshly prepared eutectic capsules in chloroform, (B) after 3 days and (C) after 8 days of storage. The samples contain 20 mg mL^−1^ GaIn eutectic alloy and different concentrations of silica (10 – 30 mg mL^−1^) nanoparticles possessing 50% SiOH on their surface.

The amount of silica nanoparticles is very important to stabilize eutectic emulsion. As shown in Fig. 3, emulsions containing 20 mg mL^−1^ GaIn and 10 and 20 mg mL^−1^ of SiO_2_ particles possessing 50% SiOH on their surface are very unstable to sedimentation even after 3 days of storage at ambient conditions. Increasing the concentration of silica nanoparticles to 30 mg mL^−1^ results in a more dense silica shell protecting and stabilizing the eutectic droplets in chloroform (see also cryo-SEM images below).

**Fig. 4:**
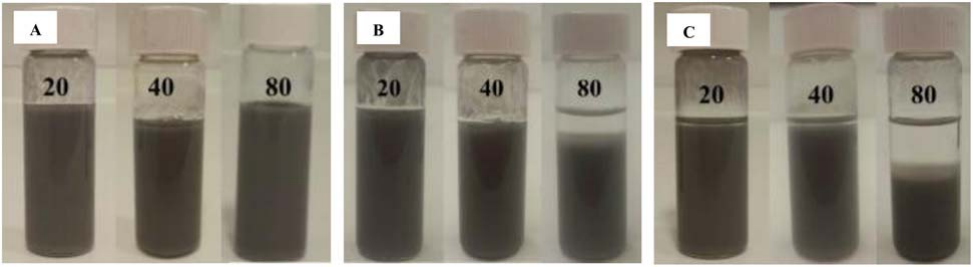
Optical photos of (A) freshly prepared, (B) after 3 days and (C) after 8 days eutectic capsules in chloroform containing different content of GaIn eutectic alloy (20 – 80 mg mL^−1^) and 30 mg mL^−1^ silica nanoparticles possessing 50% SiOH.

A similar trend is observed for samples with increased concentration of eutectic alloy in the solvent. 80 mg mL^−1^ GaIn eutectic at 30 mg mL^−1^ silica nanoparticles is unstable after 3 days of storage at ambient conditions (Fig. 4). On the contrary, 20 mg mL^−1^ GaIn alloy remains stable. An increase in particle concentration or a reduction in the volume fraction of eutectic results in increased emulsion stability. The most stable combination is 20 mg mL^−1^ GaIn eutectic in the presence of 30 mg mL^−1^ silica nanoparticles possessing 50% SiOH on their surface (average emulsion diameter 1.4 µm, polydispersity index = 9%). This combination is stable for 5 years keeping the same average diameter of 1.4 µm without gelation of GaIn capsules.

**Fig. 5:**
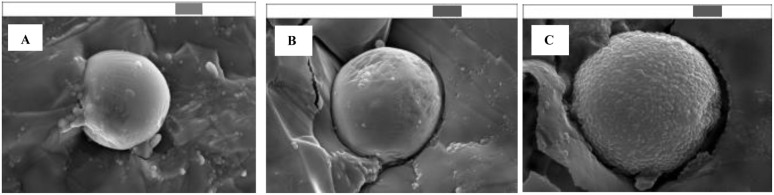
Cryo-SEM images of eutectic capsules from chloroform emulsion containing (A) 35% SiOH, (B) 50% SiOH and (C) 70% SiOH on the surface of silica nanoparticles. Silica content is 30 mg mL^−1^, GaIn eutectic content is 20 mg mL^−1^, scale bars (above) = 1 µm.

Cryo-SEM analysis of the eutectic Pickering emulsion revealed the formation of a silica nanoparticle shell around the eutectic core (Fig. 5). The increase of the SiOH content on the surface of silica leads to larger silica nanoparticles. The liquid nature of the inner encapsulated core was confirmed by mechanical destruction of the capsules with a scalpel before deposition of a platinum layer for SEM imaging (Fig. S6).

**Fig. 6:**
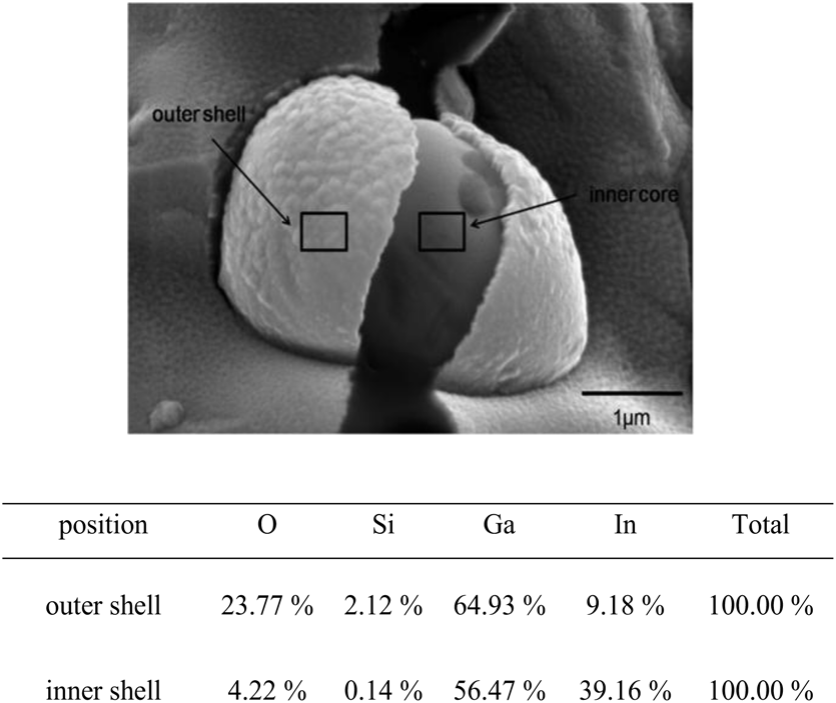
Cryo-SEM image of broken eutectic capsule in chloroform containing 20 mg mL^−1^ GaIn and 30 mg mL^−1^ silica nanoparticles possessing 50% SiOH on their surface. Below: elemental analysis of outer capsule shell and inner capsule core (in wt%).

Cryo-SEM of a broken eutectic capsule made in chloroform (Fig. 6) clearly proves the formation of a silica particle shell (thickness 80 nm) around the GaIn core. EDX analysis of the outer shell indicates the presence of silica together with Ga_2_O_3_.^12^ The inner core contains only traces of silicon.

The outer shell of eutectic capsules contains a high amount of oxygen, silicon and gallium. The inner core presents a low amount of oxygen, almost no silicon and a high indium content. The elemental analysis demonstrates oxidated Ga in the silica shell while In does not penetrate into the shell. Considering oxygen in silica, 54.98 wt% of Ga in the outer shell is in Ga oxide and hydroxides forms resulted from ultrasonic synthesis of the capsules in open air conditions. The presence of Ga and In in elemental form in EDX results is resulted from high penetration depth of EDX rays as compared to the thickness of the silica shell.

**Fig. 7:**
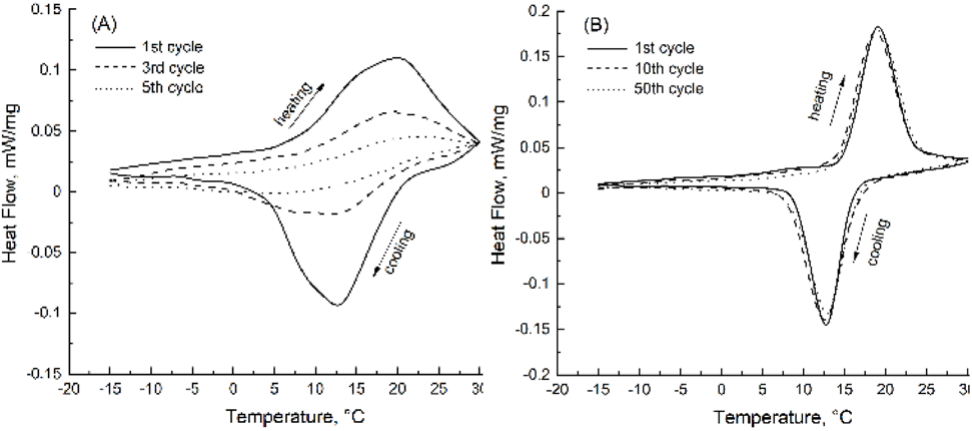
DSC of (A) bulk GaIn eutectic and (B) eutectic capsules from chloroform Pickering emulsion containing 20 mg mL^−1^ GaIn and 30 mg mL^−1^ silica particles possessing 50% SiOH on the surface.

DSC analysis of bulk and encapsulated GaIn demonstrated a considerable difference in the thermal behaviour of encapsulated eutectic (Fig. 7). A two-step solidification process was found in the cooling curve for bulk GaIn alloy. One broad solidification peak was observed between +3 °C and +20 °C (Fig. 7a, latent heat 71.5 J g^−1^). This peak indicates the formation of the mixture of stable α-GaIn phase together with metastable β-GaIn, δ-GaIn and γ-GaIn phases.^27^ In the heating curve, the broad endothermic peak can be found between +10 °C and +30 °C which corresponds to the same GaIn eutectic phases. Subsequent DSC cycling curves demonstrate degradation of heating and cooling peaks for bulk GaIn eutectic, which result from incongruent separation of eutectic components. On the contrary, encapsulation of liquid GaIn metal *via* Pickering emulsion formation revealed a sharp peak at +13 °C (cooling cycle) and at +21 °C (heating cycle), stable after >50 heating/cooling cycles (Fig. 7b, latent heat 62.2 J g^−1^). These peaks correspond to the formation of a stable α-GaIn phase. The silica particle shell stabilises melting and crystallisation of the eutectic because of the spatial confinement of GaIn eutectic. A similar stabilisation effect of the nanoconfinement of inorganic crystallohydrate PCMs was shown for other Pickering emulsions.^28^ A lower latent heat of encapsulated GaIn (62.2 J g^−1^) compared to bulk GaIn (71.7 J g^−1^) resulted from the addition of the silica shell to the overall capsule weight and partial diffusion of Ga into the shell. Latent heat of eutectic is considerably lower than in organic PCMs (150 – 250 J g^−1^); however, the advantage of eutectic is high electric conductivity.

**Fig. 8:**
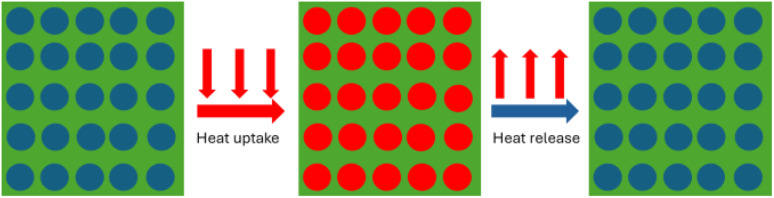
Schematic illustration of the autonomic temperature control of porous Al/Mg alloy containing GaIn eutectic capsules.

GaIn eutectic capsules demonstrated successful heat absorption and release properties after incorporation into Al/Mg alloy. The capsules fill pores and uptake extra heat from the alloy (Fig. 8). When heating is off, the capsules release extra heat into the environment.

**Fig. 9:**
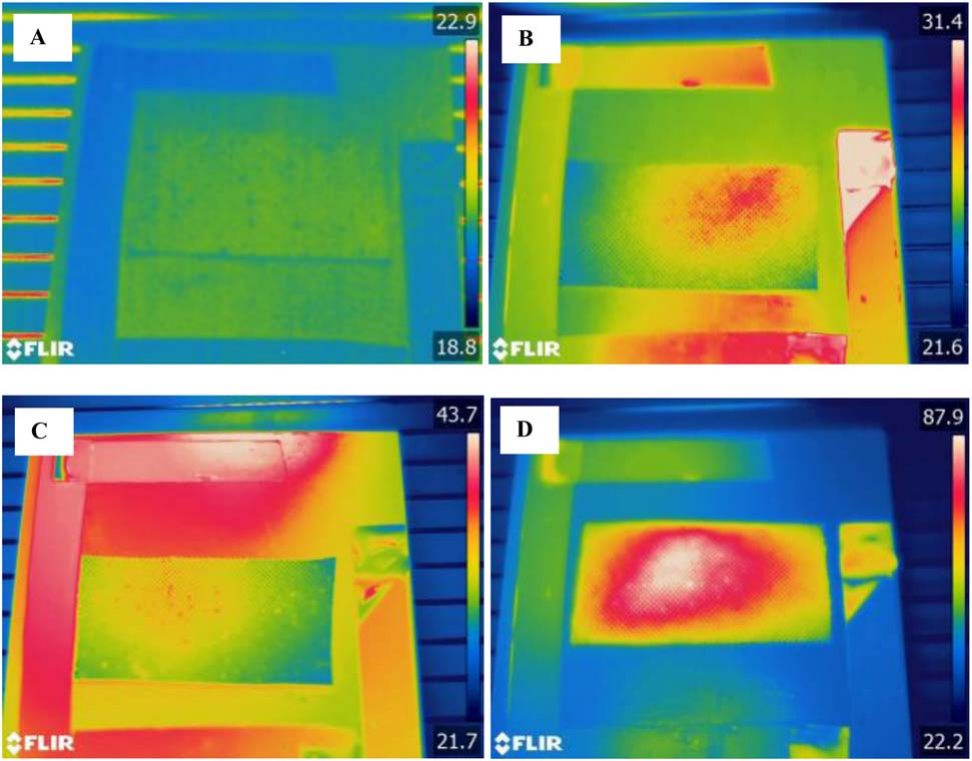
FLIR images of porous Al/Mg alloy with GaIn eutectic capsules (A) before and (B) after heating. (C) represents FLIR image of porous Al/Mg alloy without GaIn eutectic capsules before heating and (D) after heating. The colour of the images is related to the temperature/colour diagram on the right part of each image.

Al/Mg alloy was anodized to develop a porous structure on its surface.^29^ GaIn eutectic capsules were incorporated into the pores by soaking and thermal analysis of the surface was made by FLIR during heat uptake and release. Addition of 2 wt% of eutectic capsules resulted in the decrease of Al/Mg temperature after heating to 30 °C compared to the same Al/Mg sample without capsules with 82 °C increased temperature (Fig. 9). This confirms that eutectic capsules can be successfully incorporated into porous metal layers for autonomic reduction of the temperature spikes on the alloy surface. The distribution of the temperature from IR heating point (see Materials and Methods section) also demonstrates the efficiency of GaIn capsules for heat dispersion over Al/Mg surface. Al/Mg alloy with eutectic capsules effectively distributed extra heat on Al/Mg alloy surface (Fig. 9a and b) because of the high thermal conductivity of encapsuled liquid metal eutectic. Al/Mg alloy itself revealed a local heating spot (Fig. 9c and d) without heat distribution across the alloy surface. Anodised surface of Al/Mg alloy and Pickering capsule shell prevents electrochemical corrosion of Al/Mg alloy by GaIn eutectic and resulted from the absence of weight changes of the Al/Mg with encapsulated GaIn eutectic (Table S1 in SI section). On the contrary, direct incorporation into Al/Mg alloy leads to the electrochemical corrosion of the alloy.^30^

## 4 Conclusions

This research proposed the use of ultrasound for the encapsulation of liquid metal eutectic (GaIn) containing a silica shell *via* Pickering emulsion formation. The best encapsulation efficiency was found using chloroform as the continuous phase. The capsules are 1.4 µm in diameter and are stable for at least 5 years. EDX analysis of capsules confirmed an inner GaIn core and a silica outer shell. Differential scanning calorimetry indicated very good heat uptake/release performance during at least 50 thermal cycles with the latent heat of 62.2 J g^−1^ during the solid/liquid phase transition. GaIn capsules were incorporated into porous Al/Mg light weight alloy used in the aerospace industry for autonomic control of its surface temperature. Capsules demonstrated an effective decrease of the alloy surface temperature from 82 °C to 30 °C during infrared heating of the samples and increased dissipation of the heat in the Al/Mg alloy. The advantages of using liquid metal alloys as phase change materials are in their electric and thermal conductivity. These alloys can be used for autonomic thermal control in chips, batteries, robotics, photovoltaic cells and other electric systems. High toxicity of chloroform limits its application limits its application for application of eutectic capsules in green industry and can be substituted with greener solvents like ethyl lactate, which could be a subject for further investigation.

## Author contributions

B. Y., E. S., M. Q.: experimental research work, methodology and analysis of the results. H. W.: validation of the results and formal analysis. B. P. B., M. Z., D. S.: conceptualisation, writing of draft, review & editing.

## Conflicts of interest

There are no conflicts to declare.

## Supplementary Material

RA-016-D6RA02315K-s001

## Data Availability

All data generated and analysed during this study is included in this article. Supplementary information (SI) is available. See DOI: https://doi.org/10.1039/d6ra02315k.
