# Supplementary material for: Liquid metal capsules for autonomic thermal energy control
Source: RSC Adv. 2026 May 19;16(29):26740–5. doi: 10.1039/d6ra02315k (PMC13187918; doi:10.1039/d6ra02315k)
Supplement: RA-016-D6RA02315K-s001 [file RA-016-D6RA02315K-s001.pdf]

Supplementary Information (SI) for RSC Advances.
This journal is © The Royal Society of Chemistry 2026

Liquid metal capsules for autonomic thermal energy control

Supplementary Information

Bo Yu,[ab2] Hongqiang Wang,[c] Elena M. Shchukina,[a] Mikhail Zheludkevich,[d] Mathew Quarrell,[a] Bernard P. Binks,[e] Dmitry G. Shchukin[a,*]

[a]Stephenson Institute for Renewable Energy, University of Liverpool, Liverpool L69 7ZF. UK.

[b]Lanzhou Institute of Chemical Physics, Lanzhou 730000, P. R. China.

[c]School of Materials Science and Engineering, Northwestern Polytechnical University, Xi'an 710072, P. R. China.

[d] Helmholtz-Zentrum Hereon, Institute of Surface Science, Max-Planck-Straße 1, 21502 Geesthacht, Germany

[e]Department of Chemistry, University of Hull, Hull HU6 7RX. UK.

*Corresponding author: d.shchukin@liverpool.ac.uk

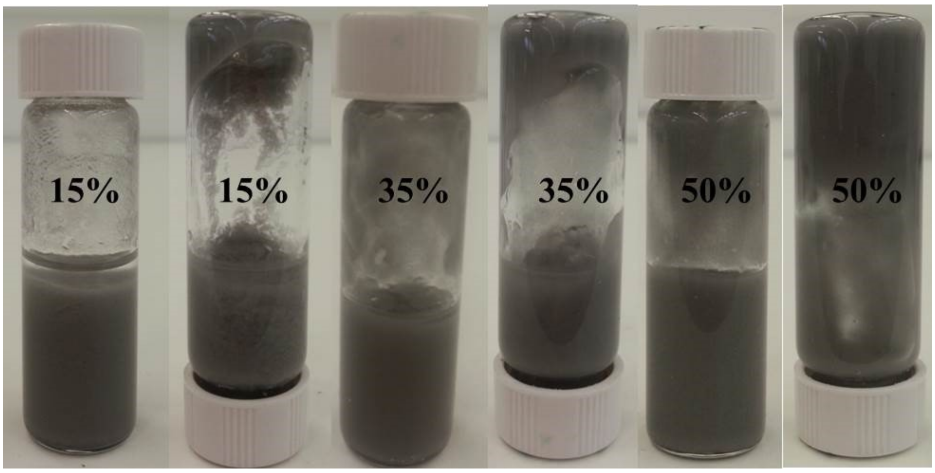

Figure S1. Photos of freshly prepared eutectic capsules in cyclohexane containing 20 mg/mL GaIn and 30 mg/mL silica nanoparticles with different %SiOH content on silica nanoparticles.

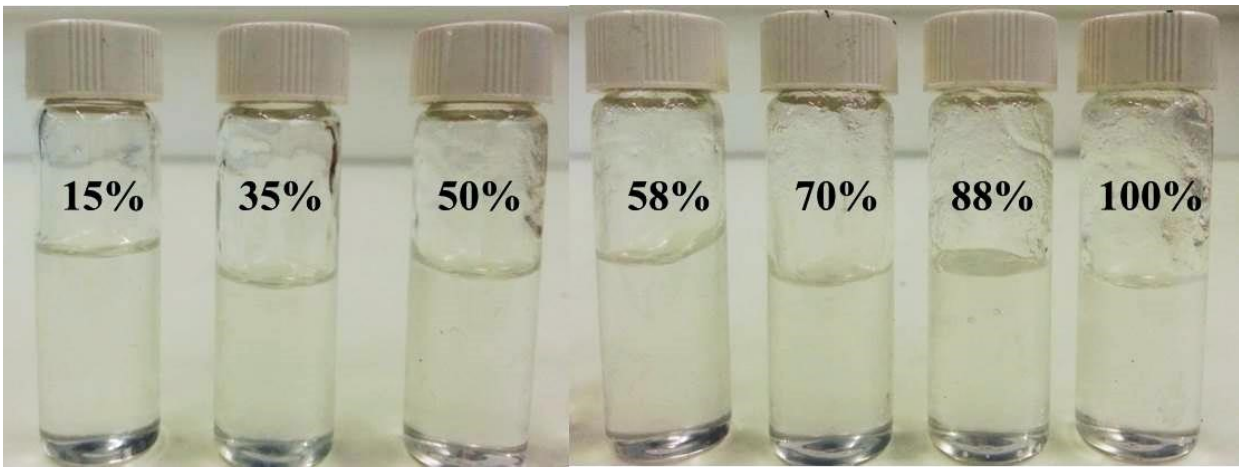

Figure S2. Photos of vessels containing 30 mg/mL silica nanoparticles in cyclohexane with different %SiOH.

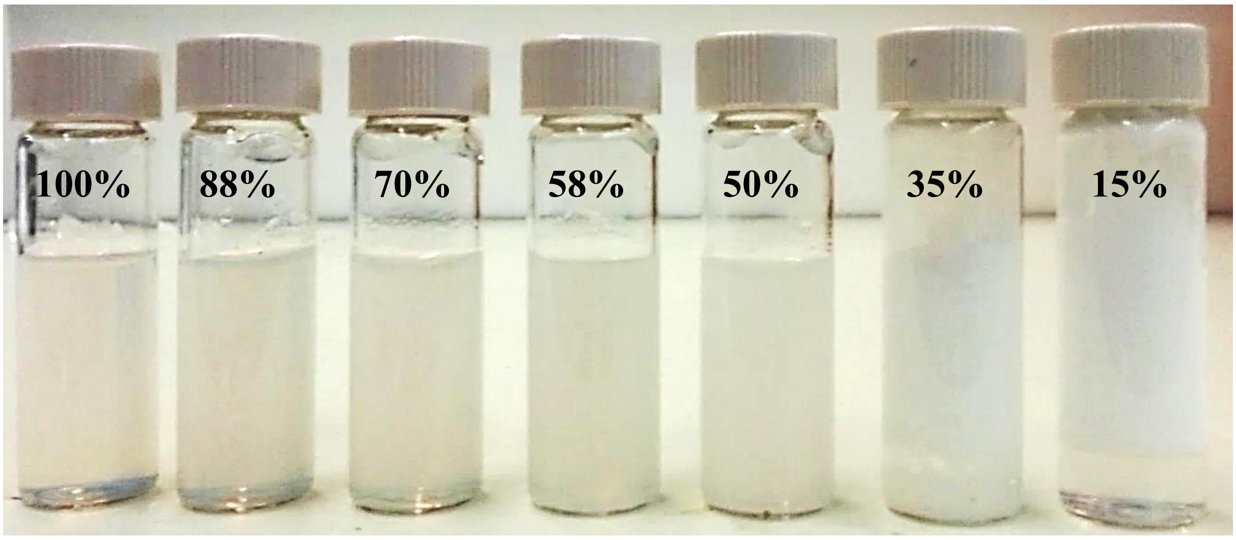

Figure S3. Photos of vessels containing 30 mg/mL silica nanoparticles with different % SiOH in water. Stable dispersions are formed from 100% to 50% SiOH groups on silica nanoparticles.

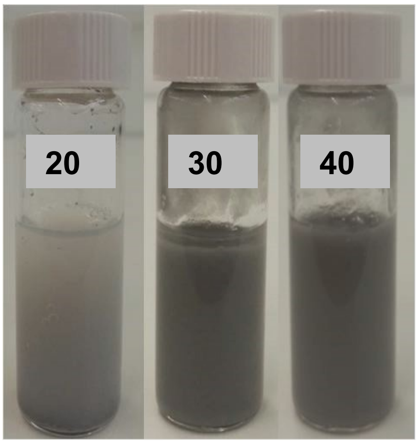

Figure S4. Photos of eutectic capsules in water containing 20 mg/mL GaIn alloy and different concentrations of 50% SiOH-modified silica nanoparticles (in mg/mL) after 8 days.

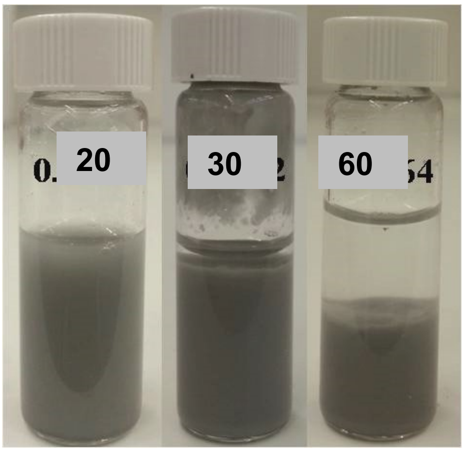

Figure S5. Photos of eutectic capsules in water containing different concentration of GaIn alloy (in mg/mL) and 30 mg/mL of 50% SiOH silica particles after 8 days.

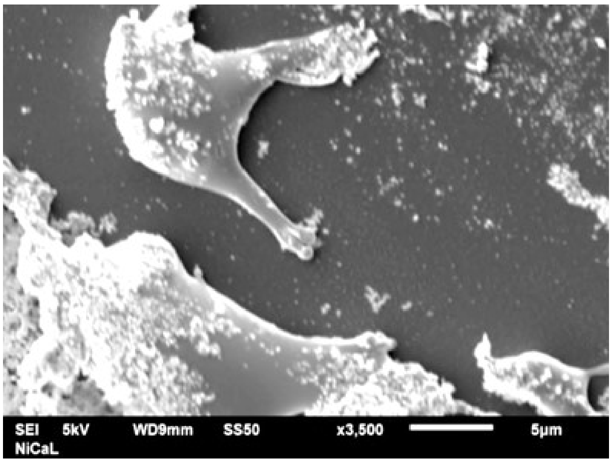

Figure S6. SEM of damaged eutectic capsules showing the liquid nature of the inner encapsulate core.

Table S1. The weight changes of Al/Mg alloy sample (2cm^2 surface and 0.3 cm thickness) with incorporated GaIn eutectic capsules during thermal cycling

Al/Mg alloy + GaIn capsules	Weight	% of weight changes
Initial sample	1.64 g	0
1st cycle	1.63 g	-0.6
5th cycle	1.63 g	-0.6
10th cycle	1.64	0
50th cycle	1.63	-0.6
